# Supplementary material for: What doesn’t kill them makes them stronger: an association between elongation factor 1-α overdominance in the sea star Pisaster ochraceus and “sea star wasting disease”
Source: PeerJ. 2016 Mar 29;4:e1876. doi: 10.7717/peerj.1876 (PMC4824914; doi:10.7717/peerj.1876)
Supplement: Supplemental Information 2 — This file provides output from model calls on raw data, sorted by model, followed by AIC model selection information. [file peerj-04-1876-s002.docx]

**Supplementary Information S2.** AIC model testing for predictive factors associated with disease in this study. These results follow general linear models (family = “binomial”) of disease status against factor and all models can be reconstructed from available R code (JPW). Interactions with Size were removed as they were insignificant in the general model and sampling in the San Juan Islands was non-random with respect to size (a target sample size of 25 for individuals <50mm radius, and 50 for individuals >50mm radius, was used in that sample of tissues).

For the model including only size (across all locations):

glm(formula = BinStatusA ~ Size.mm., family = "binomial", data = mastersub)

Deviance Residuals:

Min 1Q Median 3Q Max

-1.0251 -0.9559 -0.9144 1.4153 1.5497

Coefficients:

Estimate Std. Error z value Pr(>|z|)

(Intercept) -0.344354 0.341046 -1.01 0.313

Size.mm. -0.002492 0.003460 -0.72 0.471

(Dispersion parameter for binomial family taken to be 1)

Null deviance: 278.72 on 212 degrees of freedom

Residual deviance: 278.20 on 211 degrees of freedom

AIC: 282.2

For the model including only EF1A genotype:

glm(formula = BinStatusA ~ BinGenotype, family = "binomial",

data = mastersub)

Deviance Residuals:

Min 1Q Median 3Q Max

-1.0796 -1.0796 -0.7726 1.2784 1.6459

Coefficients:

Estimate Std. Error z value Pr(>|z|)

(Intercept) 0.5873 0.4374 1.343 0.17942

BinGenotype -0.8217 0.2999 -2.740 0.00615 **

---

Signif. codes: 0 ‘***’ 0.001 ‘**’ 0.01 ‘*’ 0.05 ‘.’ 0.1 ‘ ’ 1

(Dispersion parameter for binomial family taken to be 1)

Null deviance: 278.72 on 212 degrees of freedom

Residual deviance: 270.93 on 211 degrees of freedom

AIC: 274.93

For the model including only location as a factor:

glm(formula = BinStatusA ~ Location, family = "binomial", data = mastersub)

Deviance Residuals:

Min 1Q Median 3Q Max

-1.2201 -1.1611 -0.5106 1.1914 2.0504

Coefficients:

Estimate Std. Error z value Pr(>|z|)

(Intercept) -9.23896 1.88954 -4.890 1.01e-06 ***

Location 0.18970 0.04058 4.674 2.95e-06 ***

---

Signif. codes: 0 ‘***’ 0.001 ‘**’ 0.01 ‘*’ 0.05 ‘.’ 0.1 ‘ ’ 1

(Dispersion parameter for binomial family taken to be 1)

Null deviance: 278.72 on 212 degrees of freedom

Residual deviance: 251.33 on 211 degrees of freedom

AIC: 255.33

For the model including only interaction between genotype and location:

glm(formula = BinStatusA ~ BinGenotype * Location, family = "binomial",

data = mastersub)

Deviance Residuals:

Min 1Q Median 3Q Max

-1.2975 -1.0515 -0.4458 1.1016 2.3856

Coefficients:

Estimate Std. Error z value Pr(>|z|)

(Intercept) 4.73386 6.88566 0.687 0.4918

BinGenotype -11.27299 5.81606 -1.938 0.0526 .

Location -0.08383 0.14625 -0.573 0.5665

BinGenotype:Location 0.22230 0.12241 1.816 0.0694 .

---

Signif. codes: 0 ‘***’ 0.001 ‘**’ 0.01 ‘*’ 0.05 ‘.’ 0.1 ‘ ’ 1

(Dispersion parameter for binomial family taken to be 1)

Null deviance: 278.72 on 212 degrees of freedom

Residual deviance: 239.78 on 209 degrees of freedom

AIC: 247.78

For the model including no interaction but both genotype and location:

glm(formula = BinStatusA ~ BinGenotype + Location, family = "binomial",

data = mastersub)

Deviance Residuals:

Min 1Q Median 3Q Max

-1.3707 -0.9638 -0.5724 1.0487 1.9910

Coefficients:

Estimate Std. Error z value Pr(>|z|)

(Intercept) -8.0817 1.9324 -4.182 2.89e-05 ***

BinGenotype -0.8361 0.3181 -2.628 0.00858 **

Location 0.1902 0.0410 4.638 3.51e-06 ***

---

Signif. codes: 0 ‘***’ 0.001 ‘**’ 0.01 ‘*’ 0.05 ‘.’ 0.1 ‘ ’ 1

(Dispersion parameter for binomial family taken to be 1)

Null deviance: 278.72 on 212 degrees of freedom

Residual deviance: 244.19 on 210 degrees of freedom

AIC: 250.19

Following model summaries (note genotype significant in all models except for interaction model where power is reduced), AIC weighting performed using aicw in R to generate the following weights:

**Model Fit delta AIC weight**

No factors 280.72 32.94 5.305 x 10-8

Size (Radius) 282.20 34.42 2.531 x 10-8

EF1A Genotype 274.93 27.15 9.612 x 10-7

Region Sampled 255.33 7.554 0.0173

Region x Genotype 247.77 0.000 0.7560

Region + Genotype 250.19 2.409 0.2266
